# Human Tumor-Infiltrating Myeloid Cells: Phenotypic and Functional Diversity

**DOI:** 10.3389/fimmu.2017.00086

**Published:** 2017-02-06

**Authors:** Louise A. Elliott, Glen A. Doherty, Kieran Sheahan, Elizabeth J. Ryan

**Affiliations:** ^1^Centre for Colorectal Disease, St. Vincent’s University Hospital, School of Medicine, University College Dublin, Dublin, Ireland

**Keywords:** tumor microenvironment, monocytes, macrophages, neutrophils, myeloid derived suppressor cells, immune cell phenotyping, oncoimmunology

## Abstract

Our current understanding of human tumor-resident myeloid cells is, for the most part, based on a large body of work in murine models or studies enumerating myeloid cells in patient tumor samples using immunohistochemistry (IHC). This has led to the establishment of the theory that, by and large, tumor-resident myeloid cells are either “protumor” M2 macrophages or myeloid-derived suppressor cells (MDSC). This concept has accelerated our understanding of myeloid cells in tumor progression and enabled the elucidation of many key regulatory mechanisms involved in cell recruitment, polarization, and activation. On the other hand, this paradigm does not embrace the complexity of the tumor-resident myeloid cell phenotype (IHC can only measure 1 or 2 markers per sample) and their possible divergent function in the hostile tumor microenvironment. Here, we examine the criteria that define human tumor-infiltrating myeloid cell subsets and provide a comprehensive and critical review of human myeloid cell nomenclature in cancer. We also highlight new evidence characterizing their contribution to cancer pathogenesis based on evidence derived from clinical studies drawing comparisons with murine studies where necessary. We then review the mechanisms in which myeloid cells are regulated by tumors in humans and how these are being targeted therapeutically.

## Introduction

Cancer immunotherapy entered a new era with the introduction of immune checkpoint inhibitors. Dramatic and durable responses are now observed in patients with previously untreatable tumors. However, these remarkable outcomes are not yet achievable in all patients, and some tumor types, e.g., microsatellite stable colorectal cancers (CRC), fail to respond to these drugs. In these cases, we need further interventions to overcome the immune regulatory context of the tumor microenvironment (TME) and to harness the power of the antitumor immune response. Infiltrating myeloid cells are potent regulators of tumor-associated immune suppression, cell invasion, and metastases, and targeting of these innate immune cells may be the key to developing new immunotherapies.

**Figure 1:**
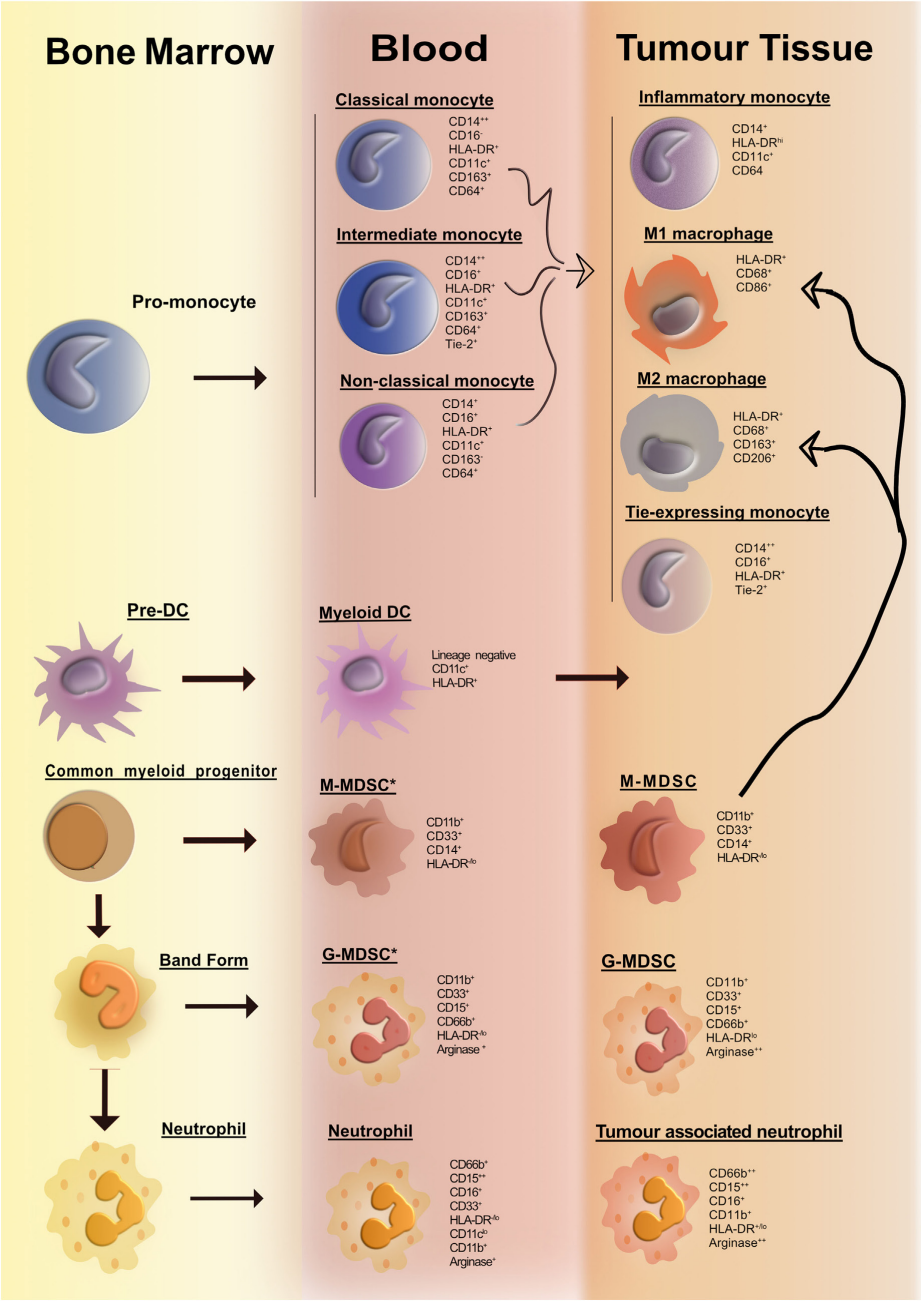
**Overview of human myeloid cells identified in the bone marrow, blood, and tissue of cancer patients**. Cancer-associated inflammation upregulates the production of myeloid cells from hematopoietic progenitors in the bone marrow. This figure illustrates the network of myeloid cells that have been identified in the blood and tumor tissue in human cancer. Cell surface markers expressed by the various myeloid cell types are listed portraying the huge degree of phenotypic similarity between the cell subsets. The thick curved black line depicts a pathway of cell differentiation that has been suggested but has not yet been proven.

In the past decade, with the advent of multiparameter flow cytometry, the identification and nomenclature of myeloid cell populations have become increasingly complex. Figure 1 illustrates our current understanding of human myeloid cell subsets and the markers that they express in the bone marrow, blood, and tissue of cancer patients. It is evident that distinct myeloid cell types may, in fact, express similar levels of certain putative lineage-specific markers, e.g., CD11b or CD68. A further important consideration is the fact that expression of many myeloid lineage markers can change upon exposure to inflammatory mediators present within tumors. One important illustration of this is the striking resemblance that human tumor-resident myeloid-derived suppressor cells (MDSCs) have with human peripheral blood neutrophils; both cell subsets are CD33^+^CD11b^+^HLA-DR^−^ and arginase-1^+^ (Arg-1). In fact, many tumors are highly infiltrated with neutrophils (as identified by their distinct morphology). Considering that no single marker can accurately differentiate between the two subsets, it is plausible to suggest that cells often designated as MDSC may also include a significant proportion of alternatively activated neutrophils. Thus, tumor-infiltrating MDSC can be viewed as a modified version of a neutrophil that has adapted to their environment and taken on an immunosuppressive function. To improve consistency among studies and minimize bias and confusion, we need to begin to move away from an oversimplified M1/M2/MDSC nomenclature. We need to consider adopting, where possible, a more complete description of myeloid cells based on a thorough assessment of lineage markers and classifications based on cell origin in combination with activation markers. Where this is not feasible, e.g., when only immunohistochemistry (IHC) analyses are practical, more careful data interpretation is needed.

A further layer of complexity in fully understanding the role of human myeloid cells in tumor biology is the degree of variation observed between individual patients and different tumor types. Progress has been made on defining key features that will enable the classification of tumors into specific subtypes allowing for better treatment stratification (e.g., HER2- or ER-positive breast cancer) (1). In CRC, it is clear that tumors displaying mismatch repair instability have higher cytotoxic lymphocyte infiltrates that correlate with a better prognosis (2). However, the relationship between myeloid cell subsets and different tumor subtypes remains to be explored.

It is difficult to ascribe precise functional roles for each of these individual myeloid cell subtypes in cancer progression. Although studies have shown that evaluation of the myeloid cell infiltrate has prognostic value (3–6), it is not yet known if these cells are a driver of the multistep process of tumorigenesis. To appreciate the importance of myeloid cells in tumor progression, we must understand how they interact within the TME. For example, dissecting the concerted interactions between myeloid cells and genetically altered cells that regulate the expression of other cancer hallmarks (cellular energetics, proliferative ability, angiogenesis, invasion, metastasis, and inflammatory signature) will help elucidate how these cells respond and evolve during tumorigenesis.

Once we understand the complexity of the mechanisms involved in myeloid regulation and how they impact on tumor progression, we can develop better therapeutic strategies that target tumor–myeloid interactions.

## Myeloid Cells in Human Cancer: Overview of Data Generated by IHC

Our understanding of the role that myeloid cells play in human cancer comes from evidence provided by retrospective cohort studies employing IHC where myeloid cells are identified by the expression of one or at most two markers, most commonly CD68 or CD163. This has led to a significant body of work demonstrating that the presence of tumor-associated macrophage (TAM) infiltrates correlate with poor patient prognosis. This is true across a variety of tumor types including breast, bladder, and ovarian cancer (3–6). Yet in other studies, a high degree of macrophage infiltration has been associated with improved patient outcome (7). Similarly, neutrophil influx as characterized by their unique morphology is associated with poor prognosis (8) and treatment failure in hepatocellular carcinoma (HCC) (9). Although these studies are informative, myeloid biology within the TME remains poorly understood, as this type of analysis cannot discriminate between different cellular activation states or give conclusive evidence regarding their function. Therefore, we need to use a more holistic approach, incorporating different technologies, e.g., CyTOF and multiparameter flow cytometry with next-generation deep sequencing to fully understand the role of different myeloid cell subsets in the regulation of antitumor immunity within different tumor environments. These types of studies will consolidate the emerging evidence that myeloid cells play an important role in tumor progression by facilitating angiogenesis and invasion (10).

## Tumor-Infiltrating Myeloid Cell Subsets

The majority of studies investigating the phenotype and function of human myeloid cells within the TME have classified these cells into one of the following categories: (i) TAMs, (ii) Tie-2-expressing monocytes (TEMs), (iii) polymorphonuclear (PMN) leukocytes (neutrophils), or (iv) MDSCs. Here, we review the different phenotypes and functions ascribed to these cell types within human tumors.

## Tumor-Associated Macrophages

Macrophages are terminally differentiated cells that reside in all tissues. They are most commonly derived from circulating monocytes and are instrumental in the orchestration of tissue homeostasis, immune surveillance, and inflammation (11).

Many studies investigating the importance of macrophages in cancer are based on the concept that two main functional phenotypes for macrophages exist, the M1 (classical) and M2 (alternative) phenotype (4, 5). The M2 phenotype is, for the most part, associated with cancer progression and worse overall survival in cancer patients (3, 4). IHC to detect cells expressing CD68, CD86 (M1), or CD163 and CD206 (M2) is frequently used to quantify and classify tumor-infiltrating macrophages (12). However, TAMs are not simply cells with these restricted “M1” or “M2” phenotypes and associated functions. In reality, the tumor macrophage compartment is more diverse and heterogeneous, with cells displaying considerable plasticity driven by environmental cues. A recent study generated a framework that showed that macrophage activation states go beyond the current M1 and M2 polarization model and represent a spectrum (13). This framework recognizes that macrophages are capable of adjusting to meet the functional requirements of their environment. One such example is the intestine, where macrophages are characterized as partially inert cells allowing them to respond to the constant exposure of dietary and commensal antigen in a controlled manner (14).

A number of research groups have examined the phenotype of tumor-infiltrating myeloid cells in different tumor types using multiparameter flow cytometry. In Table 1, we summarize a number of these recent studies. Here, we document the cell surface markers employed by each study and the main myeloid cell subset found in the tumor tissue. It is evident that there is huge variability in the myeloid cells found in the different tumor types. For example, Vasquez-Dunddel et al. found that a CD14^hi^ HLA-DR^lo^ subset resided in the tumor tissue in head and neck squamous cell carcinoma (HNSCC), whereas in gastrointestinal stromal tumors, a CD14^hi^ HLA-DR^hi^ subset was identified (15, 16). The discrepancies between these two studies may reflect the transient nature of HLA-DR expression especially when you take into consideration the very complex and variable microenvironments associated with different tumor types. However, it could be argued that these HLA-DR^lo^ cells could represent a subset of M-MDSCs (discussed in the section below) as tissue-resident macrophages are classically HLA-DR positive.

**Table 1:** **Immunophenotyping of tumor-infiltrating myeloid cells in human cancers**.

Tumor type	Markers used	Subsets identified	Reference
Bladder (urothelial carcinoma)	CD11b, HLA-DR, CD206, CD68, CCR8	CD11b^hi^HLA-DR^lo^CD206^+^CCR8^+^	(17)
Bladder	CD45, CD11b, HLA-DR, CD33, CD15	CD11b^hi^HLA-DR^hi^ CD11b^hi^CD15^hi^	(18)
Breast (ascites)	CD45, CD11b, HLA-DR, CD11c, CD14, CD16, CD1c, CD1a	CD45^+^HLADR^hi^CD11c^hi^CD16^+^CD1c^−^ CD45^+^HLADR^hi^CD11c^hi^CD16^−^CD1c^+^ (Both subsets express CD11b, CD206, and CD14)	(19)
Colorectal	CD14, CD169, CD163, CD206	CD14^+^CD169^+^CD163^+^	(20)
Colorectal	CD45, CD11b, CD11c, CD68, CD32, CD64, HLA-DR, CD80, CD86	CD45^+^CD11b^+^CD11c^+^CD68^+^CD32^+^CD64^−^HLA-DR^−^CD80^−^CD86^−^	(21)
Colorectal	CD33, HLA-DR, CD11b	CD33^+^CD11b^+^HLA-DR^−^	(22)
Gastrointestinal stromal tumor	CD11b, CD14, CD11c, CD86, CD64, CD163, HLA-DR, CD45	CD45^+^ CD11b^+^HLA-DR^hi^ CD11c^+^CD14^+^CD86^+^	(16)
Head and neck squamous cell carcinoma	CD11b, CD14, HLA-DR, CD33, CD34, CD11b, CD14, CD15	CD11b^+^CD14^+^HLA-DR^lo^ CD33^+^ CD34^+^ CD15^+^	(15)
Lung	CD11b, CD15, CD66b, MPO, arginase, CD62L, CD54, CXCR2, CCR7, CXCR3, CXCR4	CD11b^+^CD15^+^CD66b^+^ MPO^+^ Arg^+^ CD62^lo^ CD54^+^ CXCR2^lo^ CCR7^+^ CXCR3^+^ CXCR4^+^ CD11b^+^ CD15^+^	(23)
Melanoma	Lin-1^−^, CD11b, CD14, CD15	CD11b^+^ CD14^+^HLA-DR^hi^ CD11b^+^ CD14^−^ CD15^int^HLA-DR^+^ CD11b^+^ CD14^−^ CD15^hi^HLA-DR^+/lo^	(24)
Mesothelioma	CD14, CD163, CD206, HLA-DR, CD80, CD86, interleukin (IL)-4α	CD14^+^ CD163^+^ CD206^+^ HLA-DR^+^ IL-4α^+^	(25)
Ovarian (ascites)	CD45, CD11b, HLA-DR, CD11c, CD14, CD16, CD1c, CD1a	CD45^+^HLADR^hi^CD11c^hi^CD16^+^CD1c^−^ CD45^+^HLADR^hi^CD11c^hi^CD16^−^CD1c^+^ (Both subsets express CD11b, CD206 & CD14)	(19)
Ovarian (high grade)	CD2, CD3, CD4, CD15, CD45, CD16, CD19, CD33, CD133	CD45^+^CD33^+^HLA-DR^int^ CD15^−^CD16^−^	(26)
Pancreatic	Lin-1, HLA-DR, CD33, CD11b, CD15, CD14	Lin-1^−^HLA-DR^−^CD33^+^CD11b^+^CD15^+^ Lin-1^−^HLA-DR^−^CD14^+^	(27)
Pancreatic	CD45, CD14, CD15, CD11b, HLA-DR, CSF-1R	CD45^+^CD11b^+^CD14^+^HLA^−^DR^lo^ CD45^+^CD11b^+^CD15^+^	(28)

Yet a common finding also emerges that most tumor types are highly infiltrated with CD11b^+^ cells (Table 1). Not surprisingly the CD11b-expressing cell population is highly heterogeneous composed of both monocytic and granulocytic cells. Unfortunately, there is very little uniformity among the methodologies employed by these studies. A proportion of studies report that the CD11b^+^HLA-DR^+^ subset co-expresses monocyte and macrophage-associated markers CD14, CD68, CD11c (21), or M2-associated markers such as CD206 (17). However, others report that the CD11b^+^ cells represents a neutrophil-like cell based on their high CD15 expression (18, 27). Complicating matters more, in HNSCC, this subset expressed both markers CD14 and CD15, suggesting that these markers are not suitable for the delineation of macrophages and neutrophils in all tissues, particularly in the inflammatory TME (15, 29).

Interestingly, CD163^+^ and CD206^+^ macrophages derived from gastrointestinal tumors and ovarian ascites, respectively, possessed the ability to stimulate T cell responses (16, 19). As a consequence, these studies concluded that these cells were functionally equivalent to M1 or inflammatory macrophages, despite the expression of M2 like markers. Collectively, these studies reinforce the idea that the definition of a myeloid cell’s putative function based solely on their expression of cell surface markers is potentially flawed.

**Figure 2:**
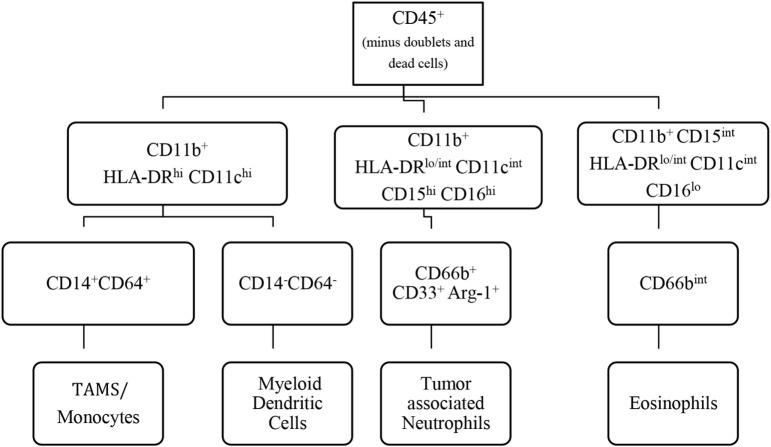
**Suggested gating strategy for the identification of tumor-infiltrating myeloid cells in humans**. To dissect the main infiltrating myeloid cells, we propose a 12 color flow cytometry panel and progressive gating strategy. Gating on the CD45^+^ population identifies the leukocyte population. Within the HLA-DR^hi^CD11c^hi^ population, tumor-associated macrophages (TAMs) can be distinguished from DCs based on CD14^+^CD64^+^ expression. The CD11b^hi^CD15^hi^ population identifies tumor-infiltrating neutrophils. CD66b is used to confirm the identity of neutrophils. Tumor-associated neutrophils (TANs) express CD33 and arginase at varying levels. It is important to note that low levels of CD64 and CD14 can be expressed on TANs, whereas TAMs can express low levels of CD15. Eosinophils are CD15^int^ and CD16^lo^. It is important to use the appropriate controls such as fluorescence minus one controls and normal or uninvolved tissue where possible.

Unfortunately, for the most part, the studies listed in Table 1 did not use sufficiently broad panels of antibodies to characterize more than one myeloid cell subset in any single data set. As a result, it is unclear how each of the individual cell subsets relates to one another within the same tumor. More importantly, the co-existence of multiple cell subsets co-expressing one or more markers cannot be ruled out. Considering tumor-infiltrating lymphocytes are an important positive prognostic and predictive marker in many cancer types, it would be interesting to determine how myeloid cell infiltrates correlate with concomitant lymphocyte infiltration (30). Differences in experimental design and antibody panels also make adequate comparisons between studies and across the different cancer types difficult. In mice, researchers have developed detailed flow cytometry panels and progressive gating strategies to accurately profile the CD45^+^ cell compartment of the tumor (31, 32). A concerted effort is needed to harmonize experimental design, gating strategies, and the cell surface markers employed so that the complete picture of the myeloid cell landscape within human tumors emerges, and we can understand how myeloid cell biology evolves with cancer progression. In Figure 2, we present a suggested flow cytometry gating strategy that allows for the identification of myeloid cells within tumor tissue.

## Tie-2-Expressing Monocytes

Tie-2-expressing monocytes that were first described in a murine model of glioma by De Palma et al. are a monocyte subset equipped with proangiogenic activity (33, 34). TEMs are predominantly part of the CD14^+^CD16^+^ monocyte subset and express elevated levels of the proangiogenic molecules vascular endothelial growth factor (VEGF), matrix metallopeptidase (MMP) 9, and insulin growth factor-1 compared to Tie-2^−^ monocytes (35, 36). As expected, there is an increased frequency of TEMs in tumor tissue (30–80%) compared to adjacent normal tissue in patients with either renal cell carcinoma (RCC) or HCC (37, 38). This suggests that TEMs accumulate in tumor tissue fueling the growth of blood vessels that help to meet the functional demand of the growing tumor. However, beyond this, our understanding of the role of TEMs in tumor pathogenesis is virtually non-existent.

Tie-2-expressing monocytes are also present in the peripheral blood of both healthy individuals and cancer patients (39, 40). It has been hypothesized that the presence of circulating TEMs could be a potential prognostic cellular biomarker for cancer. In keeping with this, TEM frequency was significantly increased in the blood of patients with HCC, enabling the differentiation of HCC from chronic liver disease (38). However, two further studies showed that TEM frequencies in the peripheral blood did not differ significantly between patients with CRC and healthy individuals (40, 41). Whether detection of circulating TEMs will have a clinically useful role in the management of cancer patients in particular settings remains to be determined in multicenter validation studies.

## PMN Leukocytes (Neutrophils)

Neutrophils are the body’s primary line of defense against invading pathogens, and their importance in cancer immunology was highlighted by the combined observations that a high blood neutrophil to lymphocyte ratio (42, 43) and tumor-infiltrating neutrophils (detected by IHC) are independent prognostic factors for tumor recurrence (4, 8, 44). The importance of neutrophils in tumor progression was further confirmed in a study employing CIBERSORT, a computational method for extrapolating leukocyte cells (45). Twenty-two immune cell subset signatures across 25 cancer histologies were examined. Remarkably a PMN-associated gene signature materialized as the most significant adverse cancer-associated prognostic factor (45). It is possible that some neutrophils are involved in preventing cancer progression, and in support of this, Amicarella et al. found that neutrophil infiltration correlated with a favorable outcome in CRC (46). Similar to the macrophage studies, these results provide correlative but not causative links between neutrophils and tumor growth or control. Therefore, the role neutrophils play in tumor biology still requires clarification in all settings and tumor types.

Neutrophils are considered a heterogeneous population of cells. Similar to the macrophage paradigm, neutrophils are reported to have dichotomous antitumor (N1) and protumor (N2) functions in mice (47). Evidence suggests that transforming growth factor (TGF)-β within the TME is largely responsible for neutrophil polarization, and inhibition of TGF-β favors the accumulation of N1 tumor-associated neutrophils (TANs). In mice, N1 and N2 TANs can only be distinguished from each other based on function. Naturally the N1-protumor phenotype secretes more immunoactive cytokines, expresses lower levels of Arg-1, and possesses greater ability to kill tumor cells (47). The majority of human studies have investigated blood neutrophil alterations in cancer patients. In HNSCC, patient’s isolated neutrophils showed reduced inducible reactive oxygen species (ROS) production and decreased spontaneous apoptosis (48). In bladder cancer, neutrophils exhibited impaired killing (49), whereas neutrophils from patients with oral cavity cancer secreted higher levels of VEGF (50) and diminished levels of soluble tumor necrosis factor (TNF)-related apoptosis-inducing ligand (51). Although informative, future studies are needed to unravel the fundamental antitumor and protumor interactions between neutrophils and malignant cells within the TME. One interesting study identified a CD10^+^ TAN (CD11b^+^CD15^+^) predominantly located at the invasive front of CRC tumors. This subset strongly correlated with tumor budding and TGF-β expression (52). Tumor buds are a small group of cancer cells (<5) thought to have undergone epithelial mesenchymal transition (EMT) changes and are an adverse prognostic factor (53). The discovery that this subset correlates with TGF-β supports the idea that this subset may represent the N2 population. In contrast, a recent study identified an antigen-presenting cell like “hybrid neutrophil” in early-stage human lung cancer defined as CD11b^+^Arg-1^+^CD66b^+^CD15^+^HLA-DR^+^CD14^+^ (54). This subset originated from an immature progenitor in response to tumor-derived interferon (IFN)-γ and granulocyte macrophage colony-stimulating factor (GM-CSF) and stimulated antitumor T cell responses. Apart from these studies, the exact functional attributes of neutrophils within the TME have yet to be fully explored in humans. Akin to the macrophage paradigm, neutrophil function and phenotype are most likely transient, constantly changing in response to their evolving environment.

One of the most controversial issues in TAN studies is their complicated relationship with MDSCs. Both subsets share phenotypic and functional similarities, which will be further explored in the next section.

## Myeloid Derived Suppressor Cells

Myeloid-derived suppressor cells are defined by their ability to suppress T cell responses and are a heterogeneous population of immature myeloid cells. MDSC were originally characterized in tumor-bearing mice, where they are classically divided into monocytic (M) (CD11b^+^/Ly6C^+^) MDSC and PMN (CD11b^+^/Ly6G^+^) MDSC (55). The consensus is that MDSCs are hematopoietic progenitor cells generated in the bone marrow that fail to undergo terminal differentiation to mature monocytes or neutrophils before being released into the circulation (56). As a result, these cells are greatly expanded under certain pathological conditions, and as their name suggests, their highly suppressive activity is their defining feature.

In man as in mice, there are two main MDSC subsets: monocytic (CD14^+^) MDSC and PMN (CD15^+^) MDSC. Classically, human MDSCs are described as lineage negative cells that co-express CD11b and CD33 but lack HLA-DR. Additional functional markers have also been attributed to MDSCs such as Arg-1, indoleamine 2, 3-dioxygenase (IDO1) ROS, and nitric oxide synthase, which all mediate immunosuppression (56).

The complexity of human MDSC characterization in patient samples is summarized in Table 2. It is clear from the literature that human tumors exhibit a great disparity in the distribution and phenotype of both PMN-MDSC and M-MDSC. The majority of the studies report an increase in the number of MDSCs in cancer patients’ blood when compared to healthy controls; however, the frequencies and phenotype reported differ greatly. For example, in bladder cancer, Uan et al. found that nearly half of circulating monocytes expressed low levels of HLA-DR (57), whereas in breast cancer, all CD14^+^ monocytes in patients’ blood expressed low HLA-DR (58). Similarly, in non-small cell lung cancer (NSCLC), two different studies identified PMN-MDSC (CD11b^+^CD15^+^CD33^+^) as the dominant MDSC subset in the blood of patients albeit at different frequencies (59, 60). It is not known whether this variability is the result of tumor-specific derived factors or a consequence of experimental design and methodology. The accelerating interest in MDSCs accompanied by the large body of information generated has led to confusion and inconsistency in MDSC nomenclature. To help address this problem, Bronte et al. developed a logical framework for defining the heterogeneous population of MDSCs in both humans and mice providing minimal phenotypic, molecular, and functional requirements (61). This gating strategy has been optimized in the peripheral blood of patients and controls, but further validation for the analysis of tumor-infiltrating MDCS is still required. It would be valuable to combine Bronte’s strategy with the one proposed in Figure 2 to gain a clearer understanding of how the different myeloid cell types co-exist within tumors. With the incorporation of these additional lineage and activation markers, a greater appreciation for the heterogeneous nature of tumor-infiltrating leukocytes can be achieved. Bronte et al. identify three subsets of MDCS in PBMC: M-MDSC (CD14^+^ HLA-DR^lo^), e-MDSC (CD33^+^lin^−^HLA-DR^−^), and PMN-MDSC (CD14^−^CD15^+^CD11b^+^). Performing this analysis on PBMC allows for the depletion of neutrophils from the sample. As a result, it can be assumed that using the combination of markers (CD11b, CD14, and CD15), PMN-MDSC can be accurately identified. However, in tissue, density gradient centrifugation is not usually performed; as a consequence, the markers CD11b, CD14, and CD15 are not sufficient as they are also expressed by neutrophils. Our panel includes CD16 and CD66b, which are classical neutrophil markers, and using this in combination with SSC profiles and CD11b, CD14, and CD15 should confirm the identity of the cells. We also included markers CD11c and HLA-DR that can classify dendritic cells and TAMs in combination with CD14, CD64, and CD11b. This allows the parallel examination of MDSC and TAMs in one sample. Finally, in the pro-inflammatory environment of tumor tissue, granulocytes may upregulate CD14. Therefore, the use of CD14 and HLA-DR alone may not be sufficient markers to identify M-MDSC within tissue. The addition of granulocyte markers will allow the accurate detection of M-MDSC.

**Table 2:** **Phenotyping of myeloid-derived suppressor cell (MDSC) in human cancers: frequency and association with suppressive activity**.

Tumor entity	Sample type	Phenotype	Frequency, patient vs. control	Arginase activity	*In vitro* activity	Reference
Bladder	PBMC	Lin^−^HLA-DR^−^CD33^+^	1.3 vs. 1.22 (% of total PBMC)	ND	ND	(57)
	PBMC	CD14^+^HLA-DR^lo^	49 vs. 32 (% of CD14^+^ cells)	High	Yes	
Colorectal	WB	Lin^−^HLA-DR^−^CD11b^+^CD33^+^CD14^−^CD15^−^CD115^−^CD13^+^	322 vs.110 (cells/ul)	No	Yes	(5)
	Tissue	CD45^+^CD11b^+^CD33^+^	5.15 vs. 1.10 (% of CD45)	No	ND	
Colorectal	PBMC	HLA-DR^−^CD33^+^CD11b^hi^CD14^−^CD18^+^CD1a^+^	1.89 vs. 0.54 (% of total PBMC)	No	ND	(22)
	Tissue	HLA-DR^−^CD33^+^CD11b^hi^CD14^−^CD18^+^CD1a^+^	2.99 (% mononuclear cells)	No	ND	
Prostate (advanced)	PBMC	Lin^−^HLA-DR^−^CD33^+^CD11b^+^CD15^+^	ND	ND	No	(62)
		Lin^−^HLA-DR^−^CD33^+^CD11b^+^CD14^+^	ND	ND	Yes	
Breast	WB	CD33^+^HLA-DR^−^	NS	ND	ND	(58)
	WB	CD14^+^HLA-DR^−^	NS	ND	ND	
	WB	CD11b^+^HLA-DR^lo^	NS	ND	ND	
	WB	CD33^+^CD13^+^CD15^−^CD14^−^	8.8 vs. 1.56 (% CD45^+^ gate)	ND	No	
	Tissue	CD45^+^CD33^+^CD13^+^CD14^−^CD15^−^	9.17 vs. 4.08 (% CD45^+^ gate)	Indoleamine 2, 3-dioxygenase (IDO1)^+^Arg-1^+^	Yes	
Breast	WB	Immature Lin^−^HLA-DR^−^CD11b^+^CD33^+^	2.85 vs. 1.26 (% of total cells)	ND	Yes (stage IV)	(63)
Glioblastoma	PBMC	CD33^+^HLA-DR^−^CD15^+^CD14^−^	12 vs. 1 (% of total PBMC)	Arg-1^+^		(64)
Hepatocellular carcinoma (HCC)	PBMC	Lin^−^CD33^+^HLA-DR^−^	2.11 vs. 1.51 (% of total PBMC)	ND	ND	(65)
HCC	PBMC	CD14^hi^HLA-DR^lo^CD11b^hi^CD11c^hi^CD33^hi^CD15^−^	16.6 vs.4.2 (% of CD14^+^ cells)	Arg-1^+^	Yes	(66)
	Tumor	CD14^hi^HLA-DR^lo^CD11b^hi^CD11c^hi^CD33^hi^	25.9 vs. 6.4 (% of CD14^+^ cells)	ND	ND	
Head and neck squamous cell carcinoma	PBMC	CD14^+^HLA-DR^lo^CD11b^+^CD33^+^CD15^−^	37.7 vs. 6.6 (% of CD11b**^+^** cells)	Arg-1^+^	No	(15)
	Tumor	CD14^+^HLA-DR^lo^CD11b^+^CD33^+^CD15^+^	ND	Arg-1^++^	Yes	
Lung cancer	PBMC	Lin^−^HLA-DR^−^CD33^+^CD11b^+^CD15^+^	ND	ND	No	(62)
		Lin^−^HLA^_^DR^−^CD33^+^CD11b^+^CD14^+^	ND	ND	Yes	
Melanoma	PBMC	CD14^+^HLA-DR^−^STAT3^hi^	54 vs. 36 (% of monocyte)	Arg-1^+^	Yes	(67)
Melanoma	PBMC	CD14^+^HLA-DR^lo^	ND	ND	ND	(68)
Melanoma	PBMC	Lin^−^HLA-DR^−/lo^CD15^+^CD33^+^CD11b^+^	ND	Arg-1^+^	ND	(69)
		CD3^−^CD19^−^HLA-DR^lo^CD14^+^	ND	ND	ND	
NSCL	PBMC	CD16^lo^CD11b^+^CD14^−^HLA-DR^−^CD15^+^CD33^+^	1.2 vs.0.064 (% of total PBMC)	Arg-1^+^	Yes	(59)
	PBMC	CD16^lo^CD11b^+^CD14^+^HLA-DR^−^CD15^−^CD33^+^	0.009 (% of total PBMC)	NEG	ND	
NSCL	PBMC	CD11b^+^CD14^−^CD15^+^CD33^+^IL-4r^+^	24.12 vs.7.50 (% of non-lymphocytic MNC)	Arg-1^+^	ND	(60)
Ovarian	WB	Lin^−^CD45^+^CD33^+^	ND	ND	No	(26)
	Tissue	Lin-1^−^CD45^+^CD33^+^HLA-DR^int^CD15^−^CD16^lo^	ND	ND	Yes	
Ovarian	Tumor	CD11b^+^CD33^+^	ND	Arg^+^	ND	(70)
	Ascites	CD34^+^		IDO1^+^		
				Interleukin-10^+^		
Pancreatic	WB	CD15^+^CD11b^+^	68.2 vs. 37.6 (% of CD45)	ND	ND	(71)
	Tumor	CD15^+^CD33^+^CD11b^+^	66.6 (% of CD45^+^)	ND	ND	
Pancreatic	PBMC	Lin^−^CD11b^+^	1.85 vs. 0.82 (% of total PBMC)	ND	ND	(72)
Prostate	PBMC	CD14^+^HLA-DR^lo^	26 vs.0.018 (% of HLA-DR)	ND	ND	(73)
Prostate	PBMC	CD14^+^HLA-DR^lo^	4.1 vs. 30.7 (% of monocyte)	ND	Yes	(74)
Renal cell carcinoma (RCC; metastatic)	PBMC	CD14^−^CD15^+^CD11c^+^CD11b^+^CD13^+^	5.49 vs.0.23 (% of total PBMC)	ND	Yes	(75)
		CD33^+^HLA-DR^−^CD11c^+^CD11b^+^CD13^+^	5.42 vs. 0.76 (% of total PBMC)	ND	Yes	
RCC	PBMC	CD15^+^CD33^+^CD11c^+^CD66b^+^CD11b^+^VEGFR1CD62L^lo^CD16^lo^	ND	Arg-1^+^	Yes	(76)

*Tumor type in which each MDSC subset is identified in indicated*.

*ND, not determined; NS, not significant; WB, whole blood; PBMC, peripheral blood mononuclear cell*.

The expansion of PMN-MDSC is much greater in tumor-bearing mice compared to that of the monocyte subset (77). However, it is unclear whether the same finding is mirrored in humans as the majority of studies have only characterized one subset. In fact only two studies have carried out a comprehensive phenotype of both MDSC subsets present in the blood of patients with NSCLC (59) and breast cancer (58). Interestingly, both studies found higher frequencies of PMN-MDSC compared to M-MDSC although different phenotypes are reported by the two studies. The former study in NSCLC identified PMN-MDSC as CD15^+^, whereas Yu et al. found that PMN-MDSC lacked CD15 expression but were CD66b^+^.

Only a few studies have evaluated the frequency and phenotype of tumor-infiltrating MDSCs (5, 15, 22, 26, 58, 66, 71). Because of the variation in experimental design, it is hard to determine which MDSC subset dominates in the tumor tissue. In both HCC and HNSCC, MDSCs defined as CD14^+^ with low HLA-DR were detected in the tumor tissue. However, in HNSCC, the subset also co-expressed CD15. This finding emphasizes how defining these cells based on a limited number of markers does not allow accurate delineation of the origin of these cells. The systematic study of both MDSC subsets in tumor tissue and patients’ peripheral blood is urgently required to better understand how these cell populations fluctuate as the tumor progresses.

The relationship between PMN-MDSC and neutrophils is another issue that remains unclear. It has been proposed that PMN-MDSCs may be a small naturally occurring subpopulation of granulocytes (78). The observation that the frequency of circulating PMN-MDSC (CD15^hi^CD11b^hi^CD33^hi^CD14^−^) did not differ between cancer patients and healthy controls, with a low frequency of less than 1%, supports this hypothesis. In addition to using the specific markers mentioned earlier, MDSCs can also be identified using the neutrophil-associated markers, CD66b and CD15 (27, 76). For example, in RCC, MDSCs isolated from peripheral blood expressed similar levels of CD15, CD11c, and CD33 compared to neutrophils. The only phenotypic difference identified was high levels of CD11b and CD66b and low levels of CD62L and CD16 expressed by MDSCs, fitting the phenotype of an activated neutrophil (76). This study clearly demonstrates the strong phenotypic similarities between MDSC and neutrophils and should make us question the rationale for studying MDSCS as a unique entity separate to granulocytes.

In humans, one of the key defining features that can help distinguish MDSCs from mature neutrophils is their lower density. As a consequence, PMN-MDSCs can be detected in the mononuclear cell fraction of peripheral blood. Recently, a distinct low-density neutrophil (LDN) subpopulation was detected along with the expected “normal” high-density neutrophil (HDN) in the blood of cancer patients (78). Interestingly, the authors’ report that the LDN population consisted of two distinct neutrophil subsets made up of immature (band) and mature (segmented) neutrophils. Similar to the study mentioned previously, the LDN subset had a greater forward scatter and expressed higher levels of CD11b and CD66b when analyzed by flow cytometry. Intriguingly, in agreement with the N1–N2 paradigm, HDNs were capable of becoming LDNs in a TGF-β-dependent manner, a switch that was accompanied by a gain of immunosuppressive properties (78).

Recent data also demonstrate that band neutrophils possess the ability to “transdifferentiate” into monocytic cells upon recruitment to inflammatory sites. The acquisition of monocyte-like characteristics was dependent on MKK6-p38 activation driven by the pro-inflammatory cytokines such as interleukin (IL)-1β, TNF-α, and GM-CSF (79). There are significant challenges to studying MDSC including their lack of unique surface markers and the plasticity these cells exhibit, particularly in response to inflammation within the TME. A more methodical approach to identifying MDSCs needs to be established if we are to define their role in tumor progression and enable development of successful therapies targeting these cells. Furthermore, important and unresolved questions remain: Are MDSCs a separate entity to granulocytes with a unique ontogeny, phenotype, and function? Or are they simply a neutrophil that represents a different functional state induced by the TME? If so, should we reconsider the nomenclature of these cells and simplify our approach to defining the cell according to their origin?

## Recruitment and Activation of Myeloid Cells within the TME

The accumulation of genetic alterations that cause neoplasia can elicit an intrinsic inflammatory response. Complex cytokine and chemokine gradients are established that recruit and shape the leukocyte infiltrate, of which myeloid cells constitutes a large proportion. Although the key components that govern myeloid cell recruitment and activation within different TMEs are yet to be fully defined, understanding how these processes are regulated will greatly accelerate the identification of new therapeutic targets. Here, we outline the most significant findings reported to date and highlight key areas for future study.

### Mechanisms of Myeloid Cell Recruitment

Among the chemotactic factors, chemokine (C-C Motif) ligand 2 (CCL2) is considered to be a key player in the recruitment of monocytes to the tumor, and the CCL2-CCR2 axis has been proposed as a new therapeutic target (80). Inhibition of CCL2-CCR2 signaling in tumor-bearing mice blocked the recruitment of inflammatory monocytes to the site of lung metastasis and prolonged their survival (81). However, the mechanisms involved in monocyte recruitment and what subsets are preferentially recruited to the tumor are not fully known in humans. There is some evidence demonstrating that high levels of CCL2 expression by cancer cells correlates significantly with TAM infiltration of tumors and poor prognosis for patients (82–84). It is important to note that a striking heterogeneity of CCL2 expression levels in human tumors has been reported. For example, reduced levels of CCL2 were detected in liver cancer and metastatic prostate cancer when compared to normal adjacent tissue, whereas higher levels were detected in breast cancer and oral squamous cell carcinoma (85). There is conflicting evidence regarding the exact source of CCL2 within the tumor. Zhou et al. identified TANs as the primary source of CCL2 and CCL17 in HCC driving the recruitment of macrophages and CCR4^+^ Treg cells, respectively, to the tumor site (9) In contrast, Spary et al. found that fibroblast-derived CCL2 promoted the migration of monocytes in prostate cancer (86).

Also, it is not clear from these studies whether CCL2 is solely responsible for the accumulation of monocytes in the tumor. There is a great degree of redundancy and complexity within the human chemokine/chemokine receptor system, and this must be taken into account the design of therapies targeting this pathway. For example, CCL2 was found to induce CCL3 expression in both human and murine macrophages, which triggered a CCL3-CCR1 signaling cascade in monocytes, essential for efficient metastasis in a mouse model (87). At present, a monoclonal antibody directed at CCL2 is in development for the treatment of patients with advanced solid tumors (trials are listed in Table 3).

**Table 3:** **Summary of novel immunotherapies targeting myeloid cells**.

Target	Drug name	Stage of development	Clinical trial identifier	Cancer subtype	Reference
Arginase-1	CB-1158	Phase 1	NCT02903914	Solid tumor^a^	
CCL2	Carlumab (CNTO 888)	Phase 1	NCT00537368	Solid tumor	(88)
		Phase 1	NCT01204996	Solid tumor	
		Phase 2	NTC00992186	Prostate	(89)
CCR2	MLN1202	Phase 2	NCT01015560	Bone metastases	
CSF-1R	BLZP45	Preclinical		GBM	(90)
	BLZP45	Preclinical		GBM	(91)
	Colony-stimulating factor-1 neutralizing antibody	Preclinical		Pancreas	(92)
	Plexidartinib (Plx3397)	Phase 2	NCT01349036	GBM	(93)
			NCT02452424	Melanoma^b^	
	Emactuzumab (RG7155)	Phase 1	NCT01494688	Solid tumor	(94)
	Ly3022855 (IMC-CS4)	Phase 1	NCT01346358	Solid tumor	
		Phase 1	NCT02718911	Solid tumor^c^	
Indoleamine 2, 3-dioxygenase (IDO1)	Indoximod	Phase 1	NCT01191216	Solid tumor	(95)
		Phase 1/2	NCT02052648	Glioma	
		Phase 1/2	NCT02073123	Melanoma^d^	
		Phase 1/2	NCT02077881	Pancreatic	
		Phase 1b/2	NCT0246036	Non-small cell lung cancer (NSCLC)	
	IDO1 peptide vaccination		NCT01219348	NSCLC	(96)
CXCR2	Pepducin	Preclinical		Pancreatic	(97)
		Preclinical		RMS	(98)
	Reparixin	Phase 1	NCT02001974	Breast	
		Phase 2	NCT02370238	Breast	
	AZD5069	Phase 1b/2	NCT02499328	SCCHN^e^	
Interleukin (IL)-8	HuMax-IL-8 (MDX 018)	Phase 1	NCT02536469	Advanced solid tumor	

*^a^combination with nivolumab*.

*^b^combination with pembrolizumab*.

*^c^combination therapy with Durvalumab*.

*^d^combination with checkpoint inhibitors*.

*^e^combination with Durvalumab*.

Several studies have described TEMs as being CCR5, CCR4, and CXCR3 positive, which suggest that they translocate in response to CCL3, CCL4, and CCL5, which are reported to be present within the TME (99–101). Furthermore, monocytes isolated from RCC patients revealed a distinct transcriptional profile compared to healthy control monocytes with significant increases in CCL3, CCL5, and CCL20 observed in the former (102). In ovarian cancer, CXCL12 promoted the recruitment of MDSCs in a prostaglandin E_2_ (PGE_2_)-dependent manner (70). However, deciphering the exact role of other chemokines involved in the recruitment of myeloid cells and effect on local immune response remains to be properly investigated in human cancer.

The TME is highly enriched with a variety of growth factors that facilitate the bidirectional communication between tumor epithelium and tumor stroma. Multiple growth factors are now emerging as potential TME-targeted therapies, including colony-stimulating factor-1 (CSF-1). CSF-1 is a principle growth factor involved in macrophage survival, differentiation, and recruitment (103). In an experimental model, inhibition of M-CSFR signaling impaired the extravasation and recruitment of monocytes into the tumor (104), whereas overexpression of CSF-1 in wild-type mice accelerated tumor progression (105). In humans, CSF-1 is widely expressed in ovarian, breast, and, renal prostate cancer and correlates with TAM infiltration and disease progression (106–108). Currently, Plexxikon (www.plexxikon.com) among others are developing CSF-1 inhibitors for a variety of cancers (summarized in Table 3). However, the blocking of CSF-1/CSF-1R may be limited as its efficacy can be dampened with the upregulation of immune checkpoint inhibitors such as programmed death ligand-1 (PD-L1) in a pancreatic cancer model (92). Targeting PD-L1 as a standalone treatment is now FDA approved for advanced melanoma and NSCLC, albeit it is only successful in approximately 34% of patients (109). Thus, combination therapy may be a more effective approach for patients who show no or minimal response to checkpoint inhibitors. In addition to its chemotactic potential, CSF-1/CSF-1R can enhance the protumor function of TAMs by modulating their cytokine signature, enriched with VEGF, TGF-β, and MMPs. Thus, the blockade of CSF-1/CSF-1R has a dual effect that may also reprogram immunosuppressive TAMs in the TME to support a more robust cytotoxic T cell response.

The TME is also enriched with other chemokines and cytokines that influence neutrophil recruitment to the tumor including granulocyte colony-stimulating factor (G-CSF). G-CSF is a cytokine that controls the production, differentiation, and function of granulocytes. IL-17A, a cytokine enriched in the tumor tissue, fostered G-CSF-mediated neutrophilia and G-CSF-driven emergency myelopoiesis (110). Interestingly, in CRC, Th17 cells recruited CD16^+^ MPO^+^ neutrophils in an IL-8-dependent manner (46). IL-8 is abundantly expressed by both normal and cancer tissue albeit at much higher levels in the latter and is associated with a poor prognosis in RCC (92). A more recent study, however, showed that IL-8 serum concentration correlated with tumor burden and stage in numerous cancers (111). Local production of IL-8 creates a chemotactic gradient that induces the recruitment of neutrophils, monocytes, and MDSC (62) *via* the chemokine receptors CXCR1 and CXCR2 (112). In a model of rhabdomyosarcoma, the blocking of CXCR2 prevented the migration of MDSC in to the TME, and interestingly, it also increased the efficacy of PD-1 targeting antibodies (98). Considering multiple immunosuppressive drivers are at play within the TME, targeting one pathway will most likely result in the activation of an alternative compensatory pathway. Thus, the synergistic effect observed here confirms that combination therapy targeting the TME will most likely be a more effective alternative treatment strategy for cancer patients. Consequently, monoclonal antibody therapy against IL-8 is now in the pipeline as a potential complementary targeted therapy to T cell-directed antibodies (Table 3).

### Mechanisms of Myeloid-Mediated Suppression

Mounting evidence indicates that the TME can alter myeloid cells converting them into potent immunosuppressive cells. In recent years, greater efforts have been made, and now researchers are starting to investigate whether these mechanisms are at play in human cancers. For example, our group has shown that tumor-conditioned media generated from human colorectal tumor explants can modulate the phenotype and function of human monocyte-derived DC (113). It is crucial that we understand the interaction between myeloid cells and the TME for us to develop and optimize the appropriate therapeutic targets. As a result, several key pathways have been identified that are now showing promising results in clinical trials.

## Indoleamine 2, 3-Dioxygenase

Indoleamine 2, 3-dioxygenase has been identified as a significant mediator of immune suppression in the TME. IDO1 is an immunomodulatory enzyme that catalyzes the breakdown of tryptophan to kynurenine rendering effector cells inactive (114). Moreover, the production of kynurenine may induce the expansion of Foxp3^+^ regulatory T cells in certain tumors (57, 114). The molecular mechanisms involved in the regulation of IDO1 expression are still not clear, but CCL20 (114) and the transcription factor, signal transducer and activator of transcription 3 (STAT3) (58), have been implicated. Although there is no IDO1 inhibitor currently approved for use in humans by the FDA, there are a few preclinical studies emerging that have investigated the inhibition of IDO1 as a potential TME target. The inhibition of IDO1 alone has failed to suppress tumor growth. However, combinational regimes with multiple chemotherapeutics have shown promising results in several phase 1 clinical trials (Table 3). For example, two thirds of patients with refractory solid malignancies who received 200 mg indoximod per day experienced objective responses or disease stabilization (95). Another promising trial found that IDO1 targeting peptide-based vaccine in combination with standard of care chemotherapy prolonged disease stabilization in nearly 50% patients with NSCLC (96).

## Arginase-1

Arginase-1 is an enzyme that metabolizes l-arginine to l-ornithine and urea (115). l-Arginine depletion by enzymatic activity of Arg-1 is probably one of the most important mechanisms employed by MDSCs to mediate local immune suppression in the tumor (116). Several human studies have shown that MDSCs suppress autologous T cell proliferation and IFN-γ production (Table 2), and the depletion of MDSC completely reversed this inhibitory effect (5, 57, 58, 63, 64, 66, 67).

To explain the suppressive mechanisms involved, several studies confirmed that MDSCs overexpressed Arg-1 and that inhibition of arginase partially restored T cell proliferation (15, 28, 57, 67). To date, only a few studies have evaluated the suppressive function of intratumoral MDSCs in different human cancers. It is postulated that MDSCs only acquire their suppressive function when activated by the TME *in situ*. Divergent observations have been described concerning MDSC suppressive activity outside the TME. Several studies (Table 2) report that circulating MDSCs could inhibit T cell proliferation. However, all studies that directly compared the suppressive activity of circulating MDCS with that of infiltrating MDSC, only the latter exhibited significant suppressive potential (15, 26, 58, 62).

Signal transducer and activator of transcription 3 is instrumental in the regulation of myeloid cell function. Importantly, STAT3 transactivation controls critical MDSC functions including the expression of arginase. The ablation of STAT3 signaling diminished the suppressive function of M-MDSC by decreasing the enzymatic activity of Arg-1 (15). Currently, there is a phase 1 clinical trial registered that is aimed at inhibiting Arg-1 in patients with solid tumors (Table 3).

More recently, a human *in vitro* model of monocyte-derived MDSCs (mo-MDSC) was shown to inhibit natural killer cell function independent of arginase activity. The suppressive activity exerted by these monocytes was mediated by TGF-β-induced PGE_2_ (117). Overall, these finding indicate that myeloid cells can block an effective T cell response by altering the amino acid composition of the TME in favor of tumor evasion.

## PD-1/PD-L1

Targeting the PD-1/PD-L1 pathway has shown dramatic antitumor effects in clinical trials, and drugs targeting this pathway have been approved for use in many tumor types (118–121). Engagement of PD-L1 on the neoplastic cells with PD-1 on activated T cells delivers an inhibitory signal that impairs T cell proliferation. In addition, PD-1 ligation alters the metabolic profile of activated T cells by inhibiting glycolysis in favor of fatty acid oxidation preventing effector cell development (122). Interfering with PD-L1 or PD-1 can block the suppressive signal delivered to the T cell causing a reboot of the immune response. Clinical studies have shown that PD-L1 expression by tumor epithelium correlates with a positive response to PD-1/PD-L1 inhibition (123). However, it is now emerging that tumor-resident myeloid cells in some cancers also express PD-L1 (86).

Our understanding of the mechanism involved in the regulation of PD-L1 on myeloid cells is limited. In prostate cancer, only soluble factors derived from stromal cells induced PD-L1 expression on monocytes, whereas epithelial soluble factors showed no effect (86). IL-6 was identified as an important regulator of PD-L1 expression with the inhibition of STAT3 preventing the upregulation of PD-L1 (86). Despite this, IL-10 did not appear to play a role in PD-L1 regulation under these experimental conditions. In tumor-bearing mice, hypoxia-induced PD-L1 expression on MDSCs, macrophages, and tumor cells in a hypoxia-inducible factor-1α-dependent manner (124). It is clear that the regulation of PD-L1 expression is complex and that multiple regulatory pathways within the TME are at play. Thus, elucidating the mechanisms regulating PD-L1 expression in the tumor will hopefully enable the development of better companion biomarkers to predict response to therapies targeting the PD-1/PDL-1 interaction.

## Mechanisms by Which Myeloid Cell Support Tumor Cell Invasion

Beyond immune suppression, myeloid cells can provide support to neoplastic cells enabling their invasion and migration by enhancing EMT changes. The EMT process converts neoplastic cells into motile and invasive mesenchymal cells allowing them to invade the surrounding stroma and seed in distant sites. This process is essential for metastasis. Evidence is emerging that myeloid cells participate in this process at both the initiation and invasion stage (125).

In breast cancer, a GM-CSF-CCL18-positive feedback loop was identified as an important mechanism in sustaining EMT and metastasis of cancer cells. GM-CSF derived from cancer cells induced neighboring macrophages to produce CCL18, which in turn caused mesenchymal-like changes of cancer cells through the activation of NF-κβ (126). Interestingly, the authors demonstrated that GM-CSF and CCL18 were both highly expressed at the invasive front of tissue sections, which were associated with a more advanced histological grade. Also in breast cancer, a subpopulation of highly motile MENA^INV^ cancer cells co-migrated with macrophages toward blood vessels in an EGF-dependent manner. MENA expression localized in cancer cells located at metastatic sites called tumor microenvironment of metastasis. These sites positively correlated with metastatic outcome in patients. Furthermore, *in vitro* assays demonstrated that cancer cells that migrated through a layer of human endothelial cells were enriched with MENA^INV^ and that intravasation was significantly enhanced in the presence of macrophages (127). In addition, an accumulation of c-Met^+^ monocytes at the invasive tumor front was associated with vascular invasion and poor prognosis in HCC. The author reported that these monocytes produced MMP-9 in response to stromal-derived HGF-9 (128). This suggests that these cells may contribute to tumor progression *via* the degradation of the extracellular matrix supporting cancer cell invasion.

In keeping with the idea that MDSCs can promote metastasis, several studies have shown that patients with extensive metastatic tumor burden present with higher numbers of MDSCs (5, 15, 58). Furthermore, MDSC frequency decreased in response to neoadjuvant therapy (129). Several human studies have investigated the mechanism by which MDSCs can shape the tumor phenotype. Aldehyde dehydrogenase 1 (ALDH1) is an important marker of cancer stem cells (CSCs). Tumors from patients with pancreatic cancer that expressed high levels of ALDH1 were associated with poor overall survival. The same study found that *in vitro* generated mo-MDSC increased ALDH1 bright CSCs in a co-culture system (28), which was reversible by blocking STAT3.

Similarly in ovarian cancer, human MDSC isolated from the TME were capable of fostering and maintaining ALDH expression within the CSC pool (26) CD33^hi^ MDSCs stimulated the upregulation of microRNA101 in ovarian cancer cells that in turn targets cell stemness repressor gene *C*-terminal binding protein (CtBP) 2. The clinical relevance of this was demonstrated in those patients who had tumors with the highest levels of CD33^hi^ cell infiltration, and the lowest levels of CtBP2 expression experienced a shorter overall survival.

Collectively these studies show that myeloid cells appear to be educated by the TME so that they adapt a trophic role that allows the tumor to express its full neoplastic potential.

## Conclusion

Tumor-resident myeloid cells are both phenotypically and functionally diverse cells. Much remains to be understood about how they evolve and function within the TME. Most importantly how they co-operate with tumor-resident lymphocytes to regulate antitumor immunity. The recent revolution in therapeutics, targeting immune checkpoint inhibitors, demonstrates that durable and complete remission is possible when the immune system is reactivated appropriately. To hasten the progress of myeloid cell-targeted therapies to the clinics, cancer immunologists need to make a concerted effort to delineate how phenotype relates to function within the entire diverse myeloid cell lineage. The identification and targeting of the key pathways that regulate myeloid cell function will allow the design of more efficacious immunotherapies.

## Author Contributions

LE reviewed the literature and drafted the manuscript. GD, KS, and ER reviewed, revised, and approved the final version.

## Conflict of Interest Statement

The authors have no other relevant affiliations or financial involvement with any organization or entity with a financial interest in or financial conflict with the subject matter or materials discussed in the manuscript.
